# Olanzapine and Lorazepam Used in the Symptomatic Management of Excited Catatonia Secondary to Anti-N-Methyl-D-Aspartate Receptor Encephalitis

**DOI:** 10.7759/cureus.8689

**Published:** 2020-06-18

**Authors:** Namita Neerukonda, Michael Bliss, Abtin Jafroodifar, Luba Leontieva

**Affiliations:** 1 Psychiatry, State University of New York Upstate Medical University, Syracuse, USA; 2 Radiology, State University of New York Upstate Medical University, Syracuse, USA

**Keywords:** anti-nmda receptor encephalitis, catatonia, olanzapine, lorazepam

## Abstract

Anti-N-methyl-D-aspartate (NMDA) receptor encephalitis has become one of the more well-known autoimmune diseases affecting the brain and it is characterized by a multitude of progressive neuropsychiatric symptoms. The following case describes the clinical course of an 18-year-old female with excited type catatonia secondary to anti-NMDA receptor encephalitis. The patient had been brought to the ED by her parents in an acutely psychotic state characterized by profound disorganization and vivid visual hallucinations. She was admitted to psychiatry and her hospital course was significant for both retarded and excited type catatonia, autonomic instability, and sensitivity to multiple neuroleptics. Given the atypicality of her symptoms and a family history of autoimmune disease, workup for autoimmune encephalitis was performed. MRI of the pelvis showed an indeterminate ovarian mass and laboratory studies were generally unremarkable. The catatonic symptoms resolved over the course of three weeks, eventually responding to a combination of lorazepam and olanzapine. Following discharge, a cerebrospinal fluid (CSF) panel resulted with positive titers for anti-NMDA receptor antibodies. This case illustrates the need to consider autoimmune encephalitis in cases of catatonia. It also presents a case in which symptoms of anti-NMDA receptor encephalitis potentially remitted without immunotherapy or mass resection.

## Introduction

Anti-N-methyl-D-aspartate (NMDA) receptor encephalitis is a neurologic disease that was first recognized in the literature in 2007 [1]. It is characterized by antibodies to the NR1-NR2 subunit of the NMDA receptor, leading to an array of neuropsychiatric symptoms [2]. It was initially thought to be a paraneoplastic syndrome primarily associated with women with co-existing ovarian teratomas [1]. However, as the literature has expanded, studies have suggested it actually to be the second most common cause of autoimmune encephalitis [3].

Anti-NMDA receptor encephalitis often starts with a prodromal phase that can include flu-like symptoms such as headaches, nausea, or fever. The initial illness portion of the condition often includes psychiatric symptoms such as anxiety, insomnia, agitation, paranoia, hallucinations (visual and auditory), delusions, and disorganized thinking [1-2]. Catatonia has also been described in numerous case reports, with studies suggesting that it is present in approximately 42% of patients with anti-NDMA receptor encephalitis [3]. Further progression can affect cognition, speech, and memory. Many cases involve new-onset seizures, autonomic instability, and in some cases death. Due to the early presentation of psychiatric symptoms, this disease has been misdiagnosed as acute psychosis or schizophrenia, making it an important differential to consider in the evaluation of acute psychotic symptoms [4]. 

Standard treatment of anti-NMDA receptor encephalitis involves tumor resection (if present) with immunotherapy consisting of corticosteroids, intravenous immunoglobulin, and plasmapheresis with generally good clinical outcomes [5]. Currently, a clear understanding of the role psychiatric medications play in the management of anti-NMDA receptor encephalitis is lacking. Numerous case reports have detailed the use of antipsychotics and benzodiazepines to alleviate psychiatric symptoms of anti-NMDA receptor encephalitis, but this is generally in the context of standard treatment for the encephalitis [6]. Here we present a case of catatonia secondary to anti-NMDA receptor encephalitis that was symptomatically managed with lorazepam and olanzapine, while remission occurred without the use of immunotherapy or mass resection.

## Case presentation

An 18-year-old African American woman with no psychiatric history was brought to the ED by her parents due to an overnight change in behavior. They reported that she had been awake all night, acting paranoid and telling them she was seeing God. On interview she was tangential, disorganized, and appeared psychotic. She spoke primarily in the third person, with no insight into the circumstances that led to her ED presentation. Multiple family members denied any observed prodromal symptoms, only reporting that she was experiencing frequent intermittent headaches over the preceding month.

There was low suspicion by her family and boyfriend for any recent drug use. Brain MRI and laboratory studies (complete blood count, basic metabolic panel, thyroid stimulating hormone, urine drug screen) were unremarkable. Her vitals were significant for tachycardia in the 100-130 beats per minute range. Family reported a history of schizophrenia in the patient’s maternal grandmother, but given the somewhat atypical presentation and the mother’s history of autoimmune disease, specifically systemic lupus erythematous, additional workup was performed for autoimmune encephalitis. Key portions of this workup would not result until approximately two weeks later.

The patient was admitted to psychiatry and initially started on risperidone with intramuscular (IM) haloperidol as needed for agitation. On the third day of admission, she developed symptoms consistent with retarded type catatonia: withdrawn, mutism, negativism, echolalia, staring, posturing, positive grasp reflex, and stereotypy. Her Bush-Francis Catatonia Rating Scale (BFCRS) score was 21 [7]. That same day her symptoms were further complicated by a brief dystonic reaction in her neck and lower extremity. Risperidone was discontinued and a lorazepam challenge provided moderate improvement of the catatonic symptoms with complete resolution of the dystonia. Her symptoms continued to improve for the remainder of the day (BFCRS decreased to six) until the next day when she began exhibiting symptoms more consistent with excited type catatonia (stereotypy, agitation, combativeness, impulsivity). Tachycardia and intermittent urinary incontinence were present as well.

Agitation during this period was managed primarily with lorazepam (averaging 10 mg total daily) due to the ongoing catatonia, the previous dystonic reaction, and as a precaution for neuroleptic malignant syndrome (NMS) after a rise in creatine kinase (CK) to around 1,000 U/L. She did not develop a fever or rigidity, but was transferred to medicine for three days to receive IV fluids due to poor oral intake and a further rise of her CK to approximately 3,000 U/L. This was ultimately attributed to intramuscular (IM) injections and restraint use. Following resolution of the rhabdomyolysis, she was started on olanzapine 10 mg twice daily after lorazepam monotherapy failed to provide adequate symptom improvement over the previous week. Most significant was her persistent distress and agitation: striking out and hitting staff, looking scared, walking backward periodically throughout the unit, all while remaining unable to share her internal experiences.

**Figure 1:**
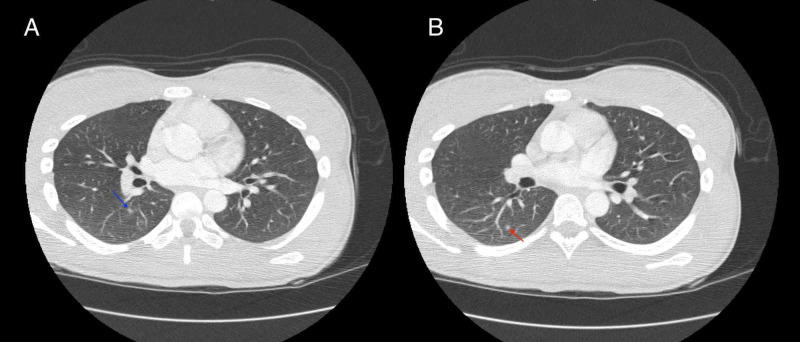
Axial CT images of the thorax. Axial CT images of the thorax demonstrate two focal areas of ground glass opacities within the superior portion of the right lower lobe. (A) The larger ground glass nodule is located posterior to the right perihilum, and measures 4.1 mm in greatest dimension (blue arrow). (B) The smaller ground glass nodule is located slightly more posterosuperiorly, and measures 3.2 mm in greatest dimension (red arrow).

**Figure 2:**
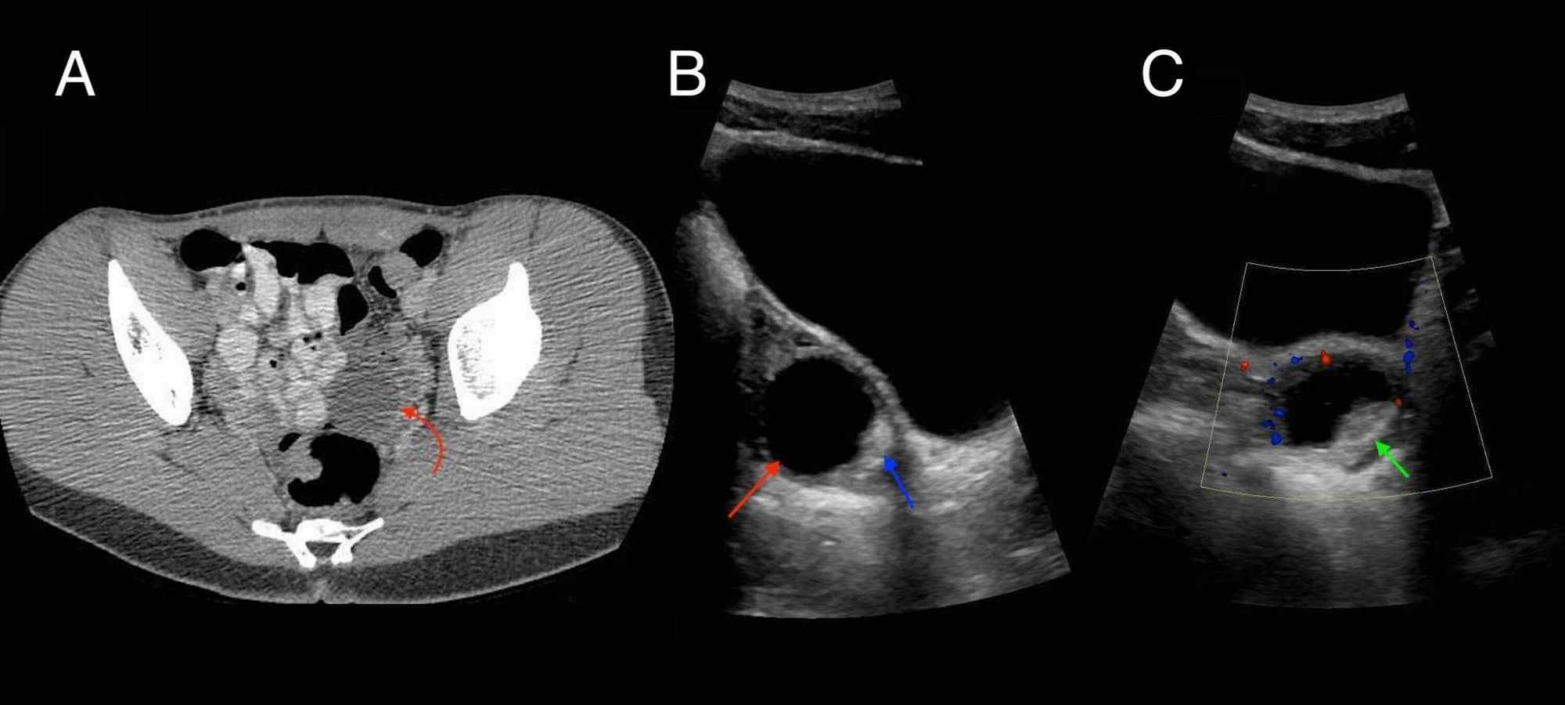
CT of abdomen and pelvis and US of pelvis. (A) Axial CT image of the abdomen and pelvis demonstrates a 3.5 cm left adnexal mass (curved red arrow) which measures fluid density centrally (approximately 4 houndsfield units). It is located anterior to the rectum and adjacent to loops of contrast-filled small bowel. (B) Sagittal ultrasound image of the pelvis without Doppler interrogation demonstrates a 3.5 cm anechoic cystic lesion within the left ovary (red arrow). The lesion demonstrates posterior acoustic enhancement. There is a smaller hyperechoic nodule along the posteroinferior margin of the lesion (blue arrow). (C) There is no definite blood flow within this nodular focus on the sagittal Doppler image (green arrow) US, ultrasound

**Figure 3:**
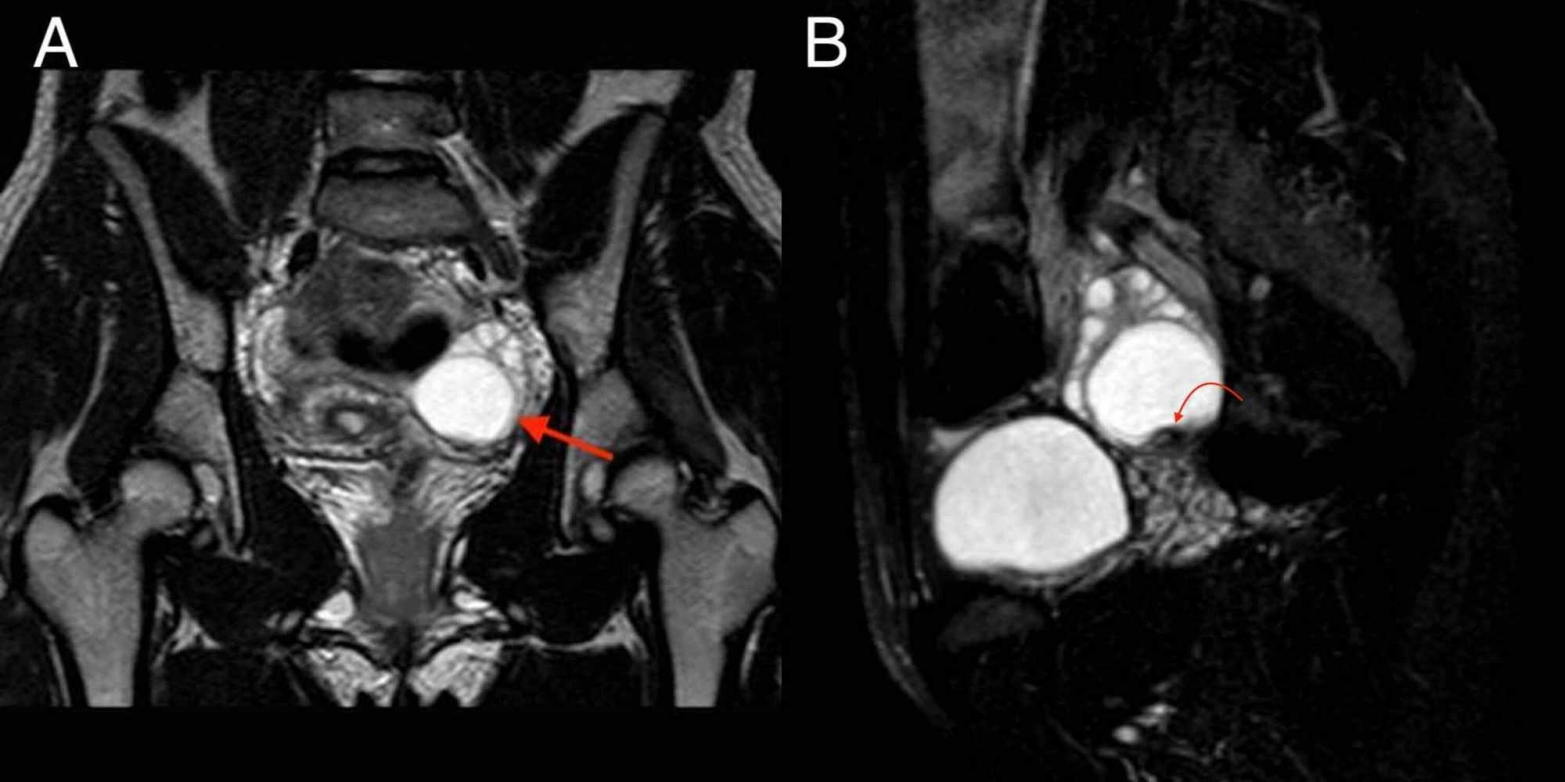
MRI of pelvis. (A) Coronal T2-weighted image through the pelvis demonstrates a round well-circumscribed lesion within the posterior left ovary, measuring 3.5 cm x 3.5 cm x 3.0 cm (red arrow). This lesion demonstrates fluid signal intensity on multiple sequences. (B) Sagittal T2-weighted image through the pelvis with fat saturation shows a small nodular hypointense focus along the caudal wall of the lesion, measuring 1.2 cm x 0.5 cm x 0.6 cm (curved red arrow).

The full serum autoimmune encephalitis panel obtained 15 days prior returned mildly elevated for voltage-gated potassium channel (VGKC) and glutamic acid decarboxylase (GAD65). These results combined with her overall clinical picture prompted further workup by neurology (Table 1). CT of the thorax was performed and showed two small ground glass opacities that indicated possible foci of infection, inflammation, or mild atelectasis (Figure 1A,B). However, CT of the abdomen and pelvis was significant because an adnexal lesion was present; that lesion was further investigated with ultrasound, which showed a 3.8 cm left adnexal cystic lesion with posterior mural nodule (Figure 2A,B). MRI of the pelvis was then suggested for further workup, and showed an ovarian mass deemed most likely to be a hemorrhagic cyst, but follow-up imaging was recommended in four to six weeks to rule out an ovarian neoplasm (Figure 3A,B). Electroencephalogram (EEG) showed excess diffuse beta activity attributed to lorazepam use. A lumbar puncture was obtained by interventional radiology, with cerebrospinal fluid (CSF) showing no significant signs of infection or inflammation. CSF antibody testing was sent to an outside laboratory and would result about one month later.

**Table 1:** Imaging studies throughout hospital admission. While MRI of the brain and EEG were largely unremarkable, a left-sided ovarian lesion was discovered and required further workup. Gynecology consultation was recommended to determine origin of the ovarian lesion as a possible hemorrhagic cyst versus an ovarian neoplasm. MRI, magnetic resonance imaging; CT, computed tomography; EEG, electroencephalogram; US, ultrasound

Date of imaging	Imaging study	Key findings
Day 4	MRI brain	Within normal limits
Day 16	CT thorax	Two ground glass nodular opacities in superior right lower lobe of lung, up to 4 mm
Day 16	CT abdomen and pelvis	3.5 cm left adnexal cystic lesion
Day 19	EEG	Excess diffuse beta activity likely secondary to medication
Day 24	US pelvis	3.8 cm left adnexal cystic lesion with posterior mural nodule
Day 25	MRI pelvis	Possible hemorrhagic cyst versus ovarian neoplasm

Once on the lorazepam and olanzapine together, her agitation gradually improved over the course of a week. When interviewed, she had no memory of the previous weeks in the hospital and denied prodromal symptoms or recent drug use. She was able to participate in psychological and cognitive assessments during the final three days of her hospitalization. The Wechsler Abbreviated Scale of Intelligence (WASI), Minnesota Multiphasic Personality Inventory - second edition (MMPI-2), and Thematic Apperception Test (TAT) selective cards (cards one, two, three, and five) were administered [8-10]. Results showed an Intelligent Quotient (IQ) of 67-69, with her overall cognitive assessment appearing inconsistent with the patient’s baseline cognitive functioning. Her MMPI-2 profile was noninterpretable due to an inconsistent pattern of responses, and her responses to TAT cards were impoverished in terms of content. The psychologists concluded that the results of the assessment were consistent with a psychosis that had not fully resolved. 

Attempts to decrease her medications prior to discharge caused swift decompositions in behavior. She was discharged on lorazepam 1 mg three times daily and olanzapine 10 mg twice daily at the request of the patient and her family, who felt she had returned to her baseline functioning. CSF testing resulting after discharge was positive for anti-NMDA receptor antibodies. Neurology saw her for follow-up six weeks later and she was reportedly doing well. She had returned to work and was off the medications because they made her tired. Montreal Cognitive Assessment (MoCA) screening showed some mild deficits (26/30) in visuospatial function, word recall, and verbal fluency [11]. She was referred to gynecology for their opinion regarding the pelvic mass, and repeat ultrasound four months later was consistent with a simple cyst. The patient did not follow-up with her outpatient psychiatry appointment. However, two years later the patient was contacted and reported complete resolution of symptoms with no return of psychotic features.

## Discussion

Anti-NMDA receptor encephalitis can be difficult to recognize and manage due to its varying array of early symptoms which have significant overlap with primary psychotic disorders such as schizophrenia. Standard management consists of immunotherapy or mass resection as curative treatment for the anti-NMDA receptor encephalitis, with psychiatric symptoms managed in conjunction using variable combinations of lorazepam, anti-psychotics, or electroconvulsive therapy [5-6]. However, various case reports suggest that clinical experiences have been mixed. 

This case is unusual in that the patient’s symptoms potentially remitted without tumor resection or any form of immunosuppression. Accounts of spontaneous remission without immunotherapy have traditionally been understood as infrequent, with recovery generally taking many months to years [12-14]. However, more recent literature suggests that there is a relapsing-remitting form that may be under-recognized [15]. Episodes of relapse and remission have been reported both in cases of anti-NMDA receptor encephalitis where immunotherapy was and was not utilized in treatment [15]. Thus, the possible relapsing-remitting nature of the disease is an important aspect to consider when managing patients with anti-NMDA receptor encephalitis.

This patient’s clinical course is not reported to suggest the use of psychiatric medication in lieu of immunotherapy treatment. It does illustrate how delays in diagnosis can occur not only from diagnostic challenges, but also from logistical factors such as the notable delay in receiving antibody testing reports. This case also highlights the need to potentially treat patients for anti-NMDA receptor encephalitis based on clinical suspicion alone, as would have occurred with this patient should her clinical course not improved, or if it developed in a manner further suggestive of anti-NMDA receptor encephalitis. Thus, while anti-NMDA receptor encephalitis remains a challenging diagnosis due to its variable clinical presentation and progression, we report a case in which olanzapine and lorazepam were effective in the symptomatic management of the disease.

## Conclusions

This case contributes to a growing body of literature describing a psychiatric symptom-predominant form of anti-NMDA receptor encephalitis which can be mistaken for primary psychotic disorders such as schizophrenia. While clinical experiences vary, our patient’s catatonic symptoms were markedly responsive to a combination of lorazepam and olanzapine. Due to the symptom resolution, anti-NMDA receptor encephalitis was not diagnosed until after discharge when the antibody report resulted weeks later. The patient’s sustained improvement at follow-up months and years later supports reports that anti-NMDA receptor encephalitis may spontaneously remit without immunotherapy or resection of an underlying neoplasm. 
